# Urinary vitronectin identifies patients with high levels of fibrosis in kidney grafts

**DOI:** 10.1007/s40620-020-00886-y

**Published:** 2020-12-04

**Authors:** Laura Carreras-Planella, David Cucchiari, Laura Cañas, Javier Juega, Marcella Franquesa, Josep Bonet, Ignacio Revuelta, Fritz Diekmann, Omar Taco, Ricardo Lauzurica, Francesc Enric Borràs

**Affiliations:** 1 REMAR-IVECAT Group, “Germans Trias i Pujol Research Institute (IGTP)” Health Science Research Institute, Campus Can RutiCarretera de Can Ruti, Camí de les Escoles s/n, 08916 Badalona, Spain; 2 Department of Cell Biology, Physiology and Immunology, Autonomous University of Barcelona, Bellaterra, Cerdanyola del Vallès, Barcelona, Spain; 3 Departament de Nefrologia i Trasplantament Renal, Institut Clínic de Nefrologia i Urologia (ICNU), Hospital Clínic de Barcelona, Barcelona, Spain; 4 Laboratori Experimental de Nefrologia i Trasplantament (LENIT), Fundació Privada Clínic Per a La Recerca Biomèdica (FCRB), Barcelona, Spain; 5 Autonomous University of Barcelona, Bellaterra, Cerdanyola del Vallès, Barcelona, Spain; 6 Nephrology Department, “Germans Trias i Pujol” University Hospital, Can Ruti Campus, Badalona, Spain; 7 ISCIII-REDinREN (RD16/0009 Feder Funds), Barcelona, Spain

**Keywords:** Urinary extracellular vesicles, Exosomes, Biomarker fibrosis, Nephrology, Noninvasive

## Abstract

**Background:**

In kidney transplantation, fibrosis represents the final and irreversible consequence of the pathogenic mechanisms that lead to graft failure, and in the late stages it irremediably precedes the loss of renal function. The invasiveness of kidney biopsy prevents this condition from being frequently monitored, while clinical data are rather unspecific. The objective of this study was to find noninvasive biomarkers of kidney rejection.

**Methods:**

We carried out proteomic analysis of the urinary Extracellular Vesicles (uEVs) from a cohort of kidney transplant recipients (*n* = 23) classified according to their biopsy-based diagnosis and clinical parameters as interstitial fibrosis and tubular atrophy (IFTA), acute cellular rejection (ACR), calcineurin inhibitors toxicity (CNIT) and normal kidney function (NKF).

**Results:**

Shotgun mass spectrometry of uEV-proteins identified differential expression of several proteins among these different groups. Up to 23 of these proteins were re-evaluated using targeted proteomics in a new independent cohort of patients (*n* = 41) classified in the same diagnostic groups. Among other results, we found a differential expression of vitronectin (VTN) in patients displaying chronic interstitial and tubular lesions (ci and ct mean > 2 according to Banff criteria). These results were further confirmed by a pilot study using enzyme-linked immunosorbent assay (ELISA).

**Conclusion:**

Urinary vitronectin levels are a potential stand-alone biomarker to monitor fibrotic changes in kidney transplant recipients in a non-invasive fashion.

**Electronic supplementary material:**

The online version of this article (10.1007/s40620-020-00886-y) contains supplementary material, which is available to authorized users.

## Introduction

Kidney transplantation is the best renal replacement therapy for patients with end-stage kidney disease in terms of survival rates [1], cost-effectiveness [2] and patients’ quality of life [3]. Decades after the first kidney transplantation, the risk of acute rejection is well controlled by the use of immunosuppressive drugs [4, 5], while chronic processes are still the target of current research [6–11]. (Chronic allograft injury or disease, which can lead to chronic allograft rejection, is a complex and multifactorial process that progressively deteriorates the graft by the appearance of renal fibrosis in an attempt of the kidney to compensate the loss of function and scar the injured tissue Chronic allograft injury or disease, which can lead to chronic allograft rejection, is a complex, multifactorial process. Fibrosis is the result of attempts by the kidney to compensate for the loss of function, leading to progressive deterioration of the graft and scarring of the injured tissue [12, 13].

The clinical parameters that are routinely used to monitor kidney function (serum creatinine levels, glomerular filtration rate and proteinuria) often fail to detect damage such as fibrotic lesions due to the compensatory mechanisms of the unaffected nephrons. Unlike the current analytical parameters, renal biopsy can identify the nature of the rejection and is currently the gold standard for diagnosis. Studies based on “protocol” biopsies have provided a great deal of information regarding the long-term evolution of the transplanted graft [14–17]. More recent developments in patient monitoring are based on identifying molecular biomarkers of rejection in kidney tissue obtained from biopsies [18–20], which have helped to reduce the frequent inter-observer variability [18, 21].

Based on Banff scoring guides, [22, 23] chronic interstitial lesions (ci) and chronic tubular lesions (ct) are the main histopathological manifestations of kidney fibrosis which may occur concomitantly with other lesions such as ischemia–reperfusion injury, rejection, infection or toxins, or it can be found in the absence of any other cause [24]. It has been shown that fibrosis accounts for more than 30% of graft losses ending in death, it is associated with worse renal function regardless of the subjacent cause [25–27] and that severe fibrosis has a prognostic value for functional decline and graft loss [28–37]. Thus, frequent monitoring of patients is urgently needed in order to detect fibrotic changes. However, renal biopsy is currently a limitation to frequent monitoring due to its invasiveness.

Instead, urine represents the ideal source of non-invasive biomarkers of graft alterations, as has been observed in the early diagnosis of rejection [38]. In particular, urinary extracellular vesicles (uEVs) are lipid-bilayered vesicles that have been studied as promising safe carriers of biomarkers. They may reflect the state of the cells that make up the excretory system, such as renal epithelial cells, glomerular podocytes, renal tubule cells, cells lining the urinary drainage system and immune infiltrating cells, thus providing a holistic view of the physiological state of the kidney through the urine [39–42].

The objective of this study was to find protein biomarkers of kidney rejection and kidney graft alterations using a bottom-up approach. We carried out proteomic analysis of uEVs of two independent cohorts of patients grouped according to their diagnosis (clinical and/or biopsy-proven). In the discovery phase, samples from the first cohort were analysed by shotgun mass spectrometry to identify candidate proteins that were differentially expressed between groups. In a second verification phase, targeted mass spectrometry was used in samples from the second cohort to confirm several proteins exhibiting differential expression levels between patients with normal kidney function and patients with renal alterations. This led to the discovery that the expression levels of uEV-associated vitronectin (VTN) were significantly increased in patients diagnosed with severe kidney fibrosis compared to those with a low/moderate degree. Vitronectin is a protein that is synthesized and secreted mainly by hepatocytes into the bloodstream, potentially resulting in extravasation into the extracellular matrix [43], thus it can be found in a variety of tissues including the kidney [44]. Moreover, urothelial cells can also synthesize vitronectin [43]. Vitronectin has a 75 kDa precursor form and a 65 kDa polypeptide isoform, with various possible glycosylations [45]. It binds, stabilizes and enhances the activity of plasminogen activator inhibitor-1 (PAI-1), [46] it is one of the main components of the extracellular matrix, participates in cell adhesion [47] and inhibits the terminal complement pathway [46].

Importantly, these results were further confirmed by enzyme-linked immunosorbent assay (ELISA) in a pilot study, pointing to the potential translationality of the biomarker to the clinical setting.

## Materials and methods

### Patients and study design

Two independent cohorts of kidney transplanted patients participated in the study: the discovery phase cohort, which included 23 patients from the Germans Trias i Pujol University Hospital (Badalona, Spain), and the verification phase cohort, that included 41 patients from the Hospital Clínic (Barcelona, Spain) (Supplementary Fig. 1a). The study population was selected according to the following inclusion criteria: (1) male or female patients older than 18 years of age; (2) ability to give informed consent; (3) absence of urinary tract infection demonstrated by the presence of leukocyturia and/or bacteriuria; (4) absence of haematuria; (5) absence of donor-specific antibody (DSA) or either active or chronic antibody-mediated rejection. Urine samples were collected immediately before biopsy. For the discovery phase, biopsies were carried out under clinical indication (i.e. when serum creatinine and proteinuria were altered). Regarding the verification phase patients, per-cause biopsies as well as protocol biopsies were considered. Histopathological diagnoses were used following the same criteria to define four groups of patients: normal kidney function (NKF), interstitial fibrosis and tubular atrophy (IFTA), acute cellular rejection (ACR) and calcineurin inhibitor toxicity (CNIT). NKF was defined by the absence of alterations in clinical parameters (serum creatinine and proteinuria) in the discovery cohort, and the absence of fibrosis and/or other findings at renal per-protocol biopsy in the verification cohort. ACR was defined by the presence of significant interstitial inflammation (> 25% of nonsclerotic cortical parenchyma, i2 or i3) and foci of moderate tubulitis (t2) [22]. Fibrosis was graded as (1) mild, less than 25% of parenchyma affected, (2) moderate, 26–50% of parenchyma affected and (3) severe, > 50% of parenchyma affected. CNIT was diagnosed after the presence of isometric tubular vacuolization and/or the presence of arteriolar wall hyalinosis was observed in patients taking calcineurin inhibitors.

This study was carried out in accordance with the Declaration of Helsinki [48] and the recommendations of the Guideline for Good Clinical Practice of the “Comitè d’Ètica de la investigació clínica de l’Hospital Universitari Germans Trias i Pujol”, which also approved the protocol. All patients were informed about the procedures and provided written informed consent to participate in the study. In order to protect human subject identity, an arbitrary code was employed for sample identification.

### Isolation of EVs from urine by size-exclusion chromatography

Morning mid-stream urine samples (approximately 75 mL) were collected right before the renal biopsy procedure or during a regular check-up visit (NKF group in the first cohort) and stored at  – 80ºC until further processing. Urine was centrifuged (600 × *g* 15 min) to eliminate cells and debris immediately after collection and frozen at  – 80ºC with the protease inhibitor AEBSF (0.138 mg/mL; Roche, Basel, Switzerland) until EV isolation. Urine was thawed overnight at 4 °C and processed following the protocol described by Puhka et al. in [49] to disrupt Tamm Horsfall polymers with some modifications. Urinary extracellular vesicles were then isolated by size-exclusion chromatography (SEC) and characterized based on the protocol described in detail by Lozano et al. [50] and Monguió-Tortajada et al. [51] with some modifications. Briefly, 40 mL of urine were centrifuged at 1800 × *g* for 10 min at 4 °C, the pellet was discarded and the supernatant was diluted 1/4 with Tris–EDTA buffer (20 mM, pH = 9). After a 90-s vortex, each sample was centrifuged at 8,000 × *g* for 15 min at 4 °C and the supernatant was concentrated using a 100 kDa cut-off Centricon filter unit. Then, 150 µL of the concentrate was loaded onto 1 mL sepharose CL-2B (Sigma-Aldrich, St. Louis, MO, USA) SEC columns and eluted with phosphate-buffered saline (PBS) to collect up to twenty 100 µL fractions (Supplementary Fig. 1b).

### Determination of uEV-enriched fractions

Protein elution from SEC was determined by reading absorbance of 2 µL of each fraction at 280 nm with Nanodrop ND-1000 (Thermo Scientific). In all cases, uEVs eluted well before the bulk of soluble proteins, which have a much smaller size (typically in fractions 5 to 8 and fractions 10–18, respectively) (Supplementary Fig. 1b). Fractions were analysed for the expression of tetraspanin-specific EV markers CD9 and CD63 by bead-based assay flow cytometry. The fractions with the highest EV marker mean fluorescence intensity (MFI) were considered to contain EV, so they were pooled together rendering a volume of approximately 300 µL of which 150 µL were used for mass spectrometry (MS) analysis.

### Mass spectrometry analysis and data analysis

#### Discovery proteomics

The proteomics analyses were performed as depicted in the workflow in Supplementary Fig. 1c. For the discovery phase, 500 µL of uEV-enriched fractions from SEC were analysed by shotgun proteomics with liquid chromatography followed by tandem mass spectrometry (LC–MS/MS). Samples were prepared for MS analysis, digested with LysC and Trypsin (Sigma-Aldrich) and injected into an Orbitrap XL. Data were analysed using the Proteome Discoverer software (v2.0, Thermo Fisher Scientific) and proteins were identified using Mascot (Matrix Science, London UK) against SwissProt human database (UniProt April 2015) [52] with a false discovery rate (FDR) of 5%.

Raw data files derived from the MS analysis in the discovery phase were processed using MaxQuant software [53] (v1.5.3.30) and SwissProt human database (UniProt, December 2015). Maximum FDR was set at 1%. Proteins identified as potential contaminants, those only identified by site or identified with a reverse sequence were discarded, as were proteins with less than 2 unique peptides. All the analyses were thereafter performed with intensity-based absolute quantification (iBAQ) values which were normalized with the EV marker ezrin.

### Targeted proteomics

Twenty-three proteins (two unique peptides for each) were selected on the basis of the results of the discovery phase, to be analysed by targeted mass spectrometry, a technique based on Selected Reaction Monitoring (SRM) [54] (see Supplementary Table 5 for the full list of the 46 peptides and sequences). A known quantity of isotypic heavily labelled standard peptides (ThermoFisher) were spiked into the samples prior to in-gel trypsin digestion for LC–MS/MS analysis. Raw data were processed using the Skyline software [55]. Ratios between the unlabelled endogenous peptide and the labelled internal standard were used to calculate the endogenous peptide quantity in each sample (fmol/sample). Measured values were normalized by the peptide abundance across samples and by endogenous ezrin for each sample. Only peptides that could be read in at least 90% of the samples by mass spectrometry were further analysed, using the mean value of the peptide pair whenever possible.

#### Enzyme-linked immunosorbent assay

Sixteen previously unanalysed urine samples from kidney-transplanted patients with different levels of biopsy-proven fibrosis grade were used in a pilot ELISA. Two millilitres of urine were concentrated to 100 µL using an Amicon Ultra of 50 kDa cut-off (Millipore, Millerica MA) at 2,000×*g* for 20 min and analysed in 96-well plates with an ELISA kit (Cloud-Clone Corporation, USA), following the protocol recommended by the manufacturer.

#### Statistical analysis of proteomics data

Enrichment analyses of Gene Ontology (GO)—Cellular Components were performed using the FunRich software [56, 57]. The FunRich software annotates Gene Ontology – Cellular Component terms based on the data from the Gene Ontology database [58, 59], HPRD [60], Entrez Gene [61] and UniProt [62]. The number of shared proteins was calculated using the online tool InteractiVenn [63]. The Perseus software [64] (v1.5.6.0 in the discovery phase or v1.6.1.3 in the verification phase) was used to perform a principal component analysis (PCA) and volcano plots. Protein expression representation and other statistical tests were performed using GraphPad Prism software (v6.0 GraphPad Software, San Diego, CA). After testing for normality, the two-sided unpaired *t*-test (parametric) or Mann–Whitney (non-parametric) were used for the comparison of the two groups of samples. In the case of multiple groups comparison, one-way ANOVA with Holm-Sidak’s multiple comparison (parametric) or Kruskall-Wallis with Dunn’s multiple comparison test (non-parametric) were performed. Hartigan test for unimodality was performed in R [65]. Finally, Gene Set Enrichment Analysis software (GSEA v3.0, Broad Institute, Cambridge, MA) [66] was used to compare enriched gene sets. The gene sets annotated by GO-Biological Process were downloaded from the GSEA molecular signatures database (MSigDB v6.2, Broad Institute, Cambridge, MA) [67]. Analyses were performed using the default parameters of GSEA, with 1,000 gene set permutations. FDR q-values < 0.25 were considered statistically significant as recommended by GSEA.

## Results

### Patients and sample collection

Two cohorts of patients (n = 23 and n = 41) were used in this study, as described in the Methods section. The patients were classified into 4 groups according to the results of the biopsy and analytical parameters: normal kidney function, interstitial fibrosis and tubular atrophy, acute cellular rejection and calcineurin inhibitors toxicity. Clinical data from the two cohorts included in the study are summarized in Tables 1 and 2. As expected, serum creatinine levels and proteinuria were higher or significantly higher in pathological groups compared to the NKF group. The histopathological results annotated using the Banff scoring system are shown in Supplementary Tables 1 and 2.

**Table 1:** Clinical parameters of the patients in the discovery cohort

Clinical parameter	NKF (n = 7)	IFTA (n = 5)	ACR (n = 6)	CNIT (n = 5)	*p*-value	Sig
Age (years) (mean ± sd)	58.1 ± 10.6	60.6 ± 8.5	49.8 ± 17.8	45.6 ± 8.6	0.147^a^	ns
Female (n (%))	4 (57.1%)	2 (40.0%)	0 (0.0%)	3 (60.0%)	0.126^b^	ns
DM (n (%))	0 (0.0%)	1 (20.0%)	1 (16.7%)	1 (20.0%)	0.672^b^	ns
Hypertension (n (%))	5 (71.4%)	5 (100.0%)	3 (50.0%)	3 (60.0%)	0.321^b^	ns
Living donor (n (%))	2 (28.6%)	1 (20.0%)	2 (33.3%)	2 (40.0%)	0.917^b^	ns
Previously transplanted (n (%))	1 (14.3%)	0 (0.0%)	1 (16.7%)	0 (0.0%)	0.635^b^	ns
*Induction treatment*						
Thymoglobulin	0	0	1	1	0.8765^b^	ns
Basiliximab	7	5	5	4		
No induction	0	0	0	0		
*Baseline immunosuppression*						
TAC-MPA	6	2	5	4	0.699^b^	ns
TAC-mTORi	0	0	0	0		
Other	1	3	1	1		
Steroid withdrawal (n (%))	0	0	0	0	–	ns
Donor age (years) (mean ± sd)	43.3 ± 9.7	51.8 ± 16.5	60.5 ± 14.3	52 ± 12.1	0.217^a^	ns
Donor sex (female, n (%))	4 (57.1%)	4 (80.0%)	3 (50.0%)	4 (80.0%)	0.620^b^	ns
Serum creatinine (mg/dL)	0.90 ± 0.11	2.20 ± 0.41	3.23 ± 1.73	2.32 ± 0.51	0.003^a^	*
Proteinuria (mg/g creatinine)	94.1 ± 49.3	672.4 ± 632.8	328.3 ± 206.6	289.6 ± 174.3	0.081^a^	ns
Months from transplantation (mean (range))	131.3 (57.8–186.6)	79.9 (15.1–252.3)	8.5 (1.1–25.2)	54.7 (0.5–238.8)	0.009^a^	*

^a^Kruskall-Wallis test was performed

^b^Chi-squared test was performed

DM, diabetes mellitus type 2; months from transplantation, months elapsed from transplantation until collection of the urine sample; Sig., significance; ns, non-significant (*p*-value > 0.01); *p-value < 0.01

**Table 2:** Clinical parameters of the patients in the verification cohort

Clinical parameter	NKF (n = 10)	IFTA (n = 11)	ACR (n = 10)	CNIT (n = 10)	*p*-value	Sig
Age (years) (mean ± sd)	46.4 ± 13.7	54.4 ± 5.9	49.5 ± 11.1	53.3 ± 15.6	0.412^a^	ns
Female (n (%))	4 (40.0%)	5 (45.5%)	3 (30.0%)	1 (10.0%)	0.325^b^	ns
DM (n (%))	7 (70.0%)	6 (54.5%)	3 (30.0%)	1 (10.0%)	0.033^b^	*
Hypertension (n (%))	9 (90.0%)	7 (63.6%)	7 (70.0%)	7 (70.0%)	0.561^b^	ns
Living donor (n (%))	3 (30.0%)	2 (18.2%)	2 (20.0%)	10 (100.0%)	< 0.001^b^	***
Previously transplanted (n (%))	0 (0.0%)	3 (27.3%)	0 (0.0%)	1 (10.0%)	0.112^b^	ns
*Induction treatment*						
Thymoglobulin	8	8	4	2	0.0647^b^	ns
Basiliximab	2	1	5	6		
No induction	0	2	1	2		
*Immunosuppression*						
TAC-MPA	7	3	5	6	0.545^b^	ns
TAC-mTORi	3	7	4	3		
Other	0	1	1	1		
Steroid withdrawal (n (%))	1	2	0	1	0.5788^b^	ns
Donor age (years) (mean ± sd)	37.8 ± 21.6	49.6 ± 9.4	48.5 ± 11.4	59.4 ± 9.1	0.010^a^	*
Donor sex (female, n (%))	7 (70.0%)	2 (18.2%)	5 (50.0%)	9 (90.0%)	0.007^b^	**
Serum creatinine (mg/dL)	1.11 ± 0.60	1.79 ± 0.75	1.86 ± 0.52	1.95 ± 0.94	0.007^a^	**
Proteinuria (mg/g creatinine)	114 ± 148	634 ± 1511	646 ± 740	156 ± 115	0.004^a^	**
Months from transplantation (mean (range))	11.5 (4.2–20.3)	27.0 (3.6–172.1)	39.7 (0.5–188.9)	47.2 (1.57–251.1)	0.246^a^	ns

^a^Kruskall-Wallis test was performed

^b^Chi-squared test was performed

DM, diabetes mellitus type 2; Steroid withdrawal, at biopsy date; months from transplantation, months elapsed from transplantation until collection of the urine sample; Sig., significance; ns, non-significant (*p*-value > 0.01); *p-value < 0.01; **p-value < 0.001

### Discovery phase

Proteomic characterization of uEV-enriched fractions.

**Fig. 1:**
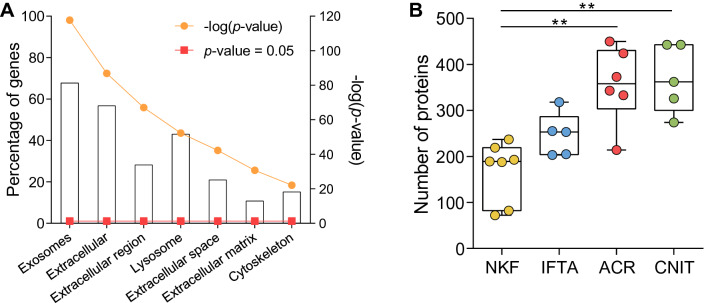
**a** Gene Ontology – Cellular Component (GOCC) analysis of proteins found in all uEV samples. Bars represent the percentage of proteins related to that GOCC in the samples. Orange circles show the -log10(*p*-value) of the enrichment, while red squares signal the significance reference *p*-value = 0.05. **b** Number of proteins found in uEV samples of each study group. NKF, normal kidney function; IFTA, interstitial fibrosis and tubular atrophy; ACR, acute cellular rejection; CNIT, calcineurin inhibitor toxicity (** *p* < 0.001)

Urinary EV-enriched fractions were analysed by their proteomic content. In the discovery phase, a total of 1,121 proteins were identified among all samples. After strict filtering of these sequences, up to 777 proteins were confidently identified, including EV-specific proteins such as Ezrin, CD9 and CD81 tetraspanins and the Annexin and 14–3-3 families in almost all samples. FunRich analysis reported that the most enriched Gene Ontology—Cellular Component (GOCC) was “Exosomes”, followed by “Extracellular”, “Extracellular region” and “Lysosome”, all with a *p*-value < 0.001 (Fig. 1a). ACR and CNIT presented a significantly higher number of proteins than the NKF group (Fig. 1b). There were 15 proteins shared by all 23 samples, which are related to “Exosomes” according to the GOCC analysis (Supplementary Fig. 2 and Supplementary Table 3).

### Alterations in grafted kidneys are reflected in uEV proteomic analysis

**Fig. 2:**
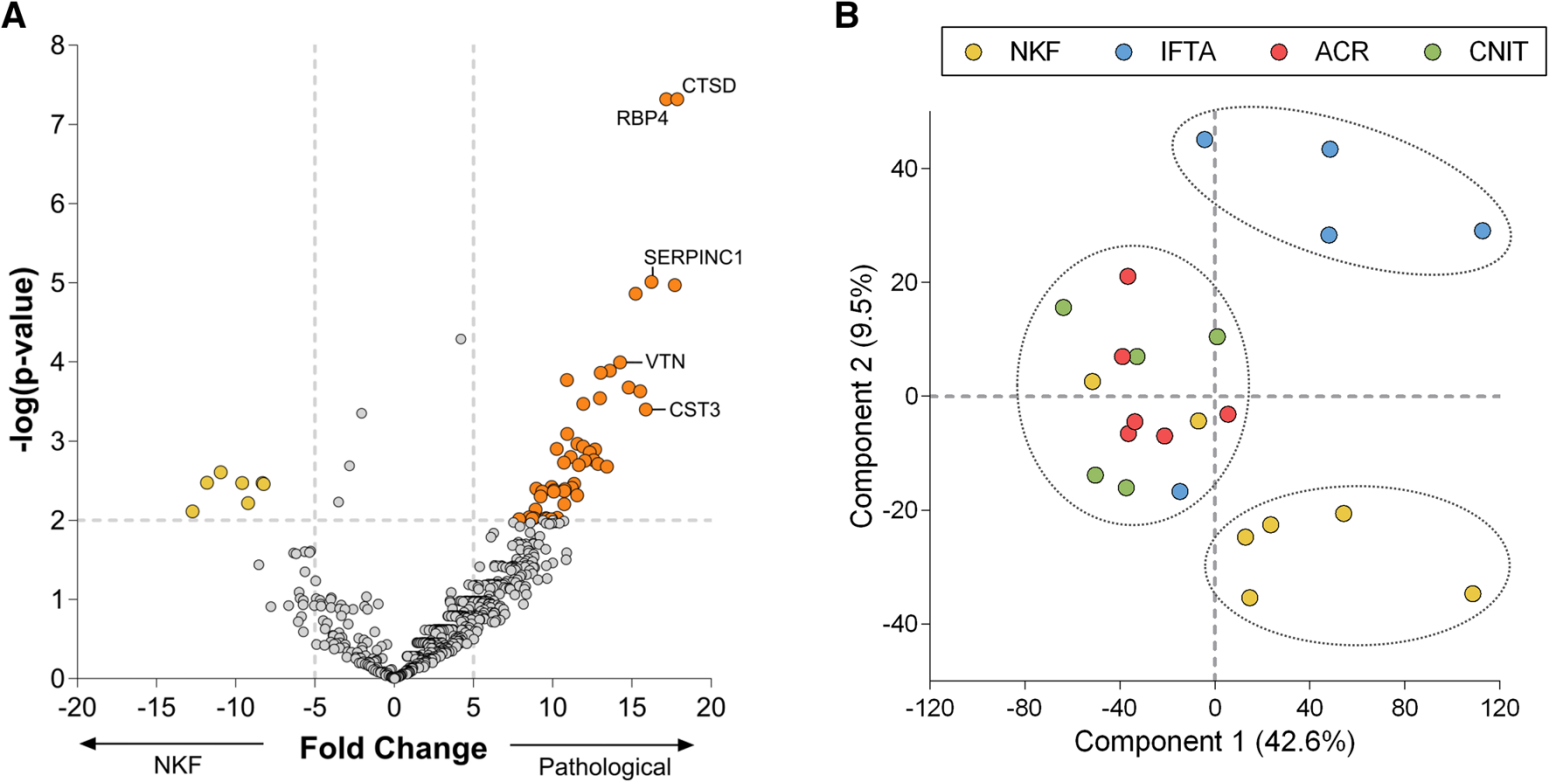
Differentially expressed proteins between pathological and NKF groups. **a** Volcano plot showing proteins significantly more expressed in pathological (IFTA, ACR and CNIT; to the right) and NKF (to the left) uEV samples. Each dot represents a protein. Y-axis represents –log(*p*-value), where significant *p*-value = 0.01 is indicated by a horizontal dashed grey line. Significant proteins with a fold change > 5 or < -5 (indicated by vertical dashed grey lines on the x-axis) are shown with bigger darker circles. The proteins investigated later on are labelled with their gene name. **b** Principal component analysis (PCA) that shows the distribution of samples according to Components 1 (which explains 42.6% of the variability among samples, x-axis) and 2 (which explains 9.5% of the variability among samples, y-axis). Samples were coloured according to their group. Dashed lines circle samples clustering. CTSD, Cathepsin D; RBP4, retinol binding protein 4; VTN, vitronectin; CST3, cystatin C; SERPINC1, antithrombin

A volcano plot was performed to visualize differentially expressed proteins between the pathological groups and the NKF group (Fig. 2a). Only proteins with *p*-value < 0.01 and fold change > 5 or <  – 5 were considered significantly different. Seven proteins were over-expressed in the NKF group, while a total of 48 proteins were over-expressed in the pathological group. These included cathepsin D (CTSD), retinol binding protein 4 (RBP4), antithrombin (SERPINC1, previously known as antithrombin III), vitronectin and cystatin-C (CST3) (the full list of differentially expressed proteins is shown in Supplementary Table 4).

Then, a PCA was performed to reveal clustering of samples according to their proteomic profile. As shown in Fig. 2b, most NKF samples (5 out of 7) were clearly segregated from the rest of the samples. Interestingly, most IFTA samples (4 out of 5) were also segregated from ACR and CNIT, which constituted the third cluster.

### Gene set enrichment analysis in IFTA

**Fig. 3:**
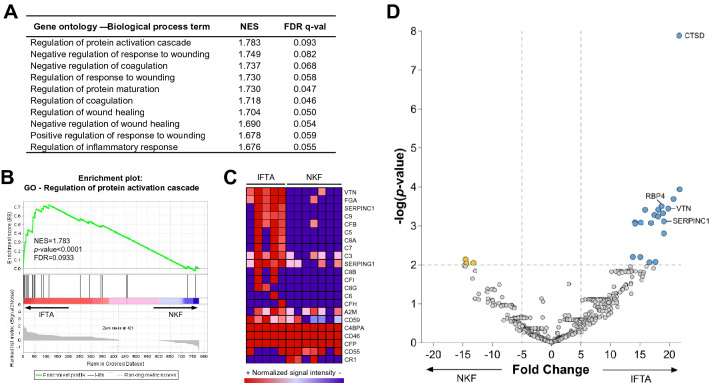
The uEV proteome shows significant differences between IFTA and NKF. **a, b** The differences between IFTA and NKF were investigated using the Gene Set Enrichment Analysis (GSEA) software, under the conception that each gene corresponds to a protein. **a** List of the ten most enriched Gene Ontology – Biological Process (GO-BP) gene sets in IFTA compared to the NKF group. **b** GSEA Enrichment plot of the GO-BP “Regulation of protein activation cascade”. Shown on the x-axis is the rank order of the IFTA genes from the most up-regulated (position 1) to the most down-regulated (position 777) compared to NKF. The “barcode” indicates the position of the genes of the mentioned gene set in this rank. The y-axis shows the enrichment score (ES) which is higher when genes found in that pathway are up-regulated in IFTA, Normalized enrichment score (NES). **c** Heat map of the expression of proteins of the GO-BP “Regulation of protein activation cascade” in IFTA and NKF uEVs. **d** Volcano plot showing proteins significantly more expressed in IFTA (to the right) and NKF (to the left) uEV samples by targeted mass spectrometry. The proteins investigated later on are labelled with their gene name. CTSD, Cathepsin D; RBP4, retinol binding protein 4; VTN, vitronectin; SERPINC1, antithrombin

Given the results of the PCA, we further focused on the differences between the NKF and IFTA groups. According to GSEA results, 46 gene sets were significantly enriched (nominal *p*-value < 0.01) in the IFTA group as were 26 gene sets in the NKF group (Fig. 3a). The most enriched Biological Process term according to Gene Ontology in IFTA compared to NKF was Regulation of protein activation cascade (Fig. 3b). This gene set is composed of 35 genes, 22 of which were found in IFTA samples. Importantly, the three most expressed genes of this gene set were VTN, fibrinogen alpha chain (FGA) and SERPINC1 and other proteins related to the Complement system (Fig. 3c). A volcano plot was also performed to visualize the proteins with significantly different expression between NKF and IFTA groups, where CTSD and RBP4 were amongst the significantly different proteins, as were VTN and SERPINC1 (Fig. 3d). This information, together with the previous analyses were used to select the 23 candidate proteins for the verification phase.

### Verification phase

Candidate protein verification: vitronectin is significantly more expressed in high grade fibrosis samples.

In a second cohort of patients, twenty-three proteins selected as candidates for verification were analysed by targeted proteomics. From a total of 41 samples of the second cohort, four samples were discarded from the analysis either because of technical error or lack of peptide signal detection in the targeted proteomics analysis (final n = 37). Only peptides that were detected in at least 90% of the samples were analysed, which resulted in 15 proteins.

**Fig. 4:**
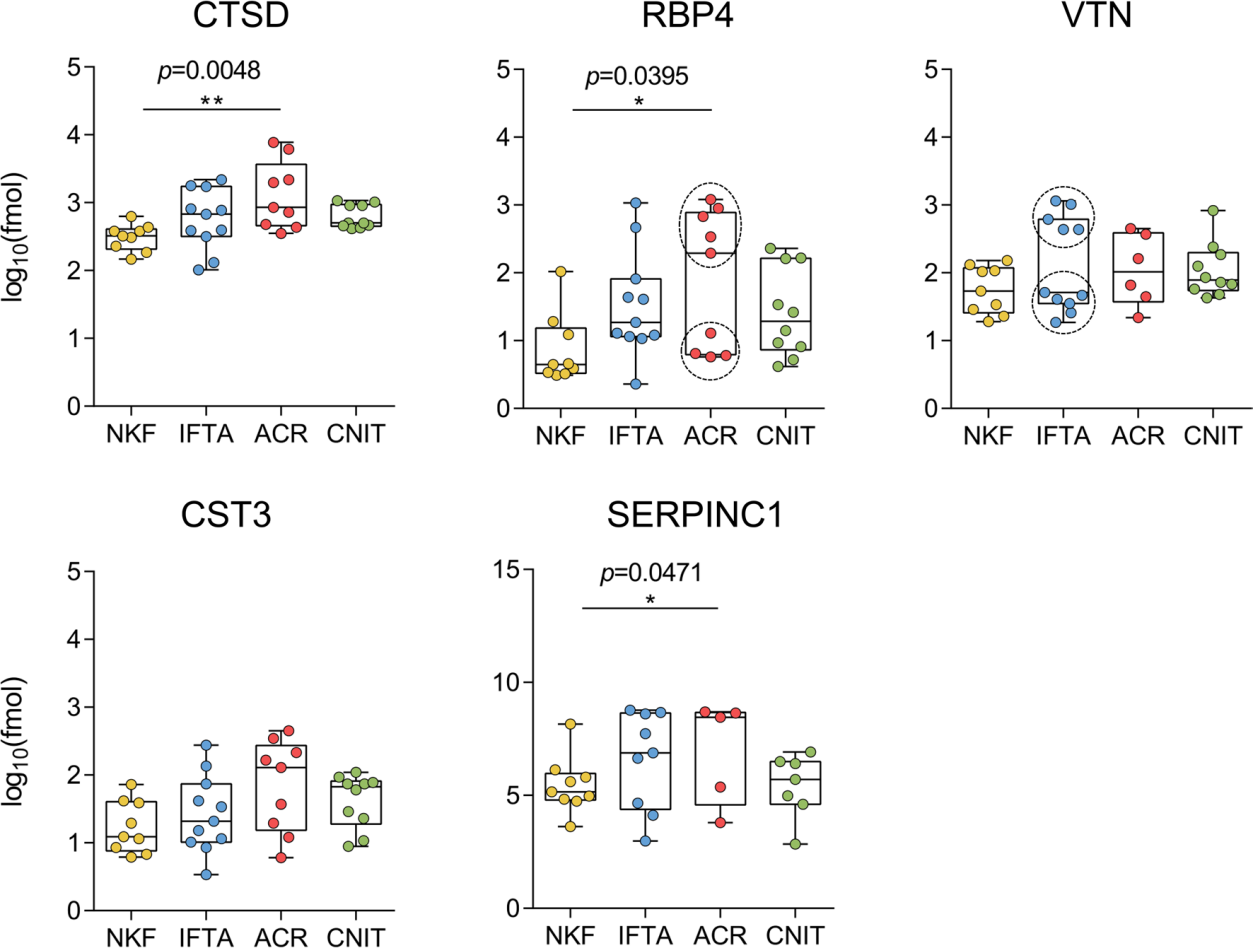
Protein expression in uEVs of each group of the verification cohort. Protein levels were measured by targeted mass spectrometry. In RBP4 and VTN, dashed circles were used to highlight the binomial distribution of individual samples in ACR and IFTA, respectively. Boxplots show the mean of each group and whiskers show minimum to maximum. CTSD, Cathepsin D; RBP4, retinol binding protein 4; VTN, vitronectin; CST3, cystatin C; SERPINC1, antithrombin

**Fig. 5:**
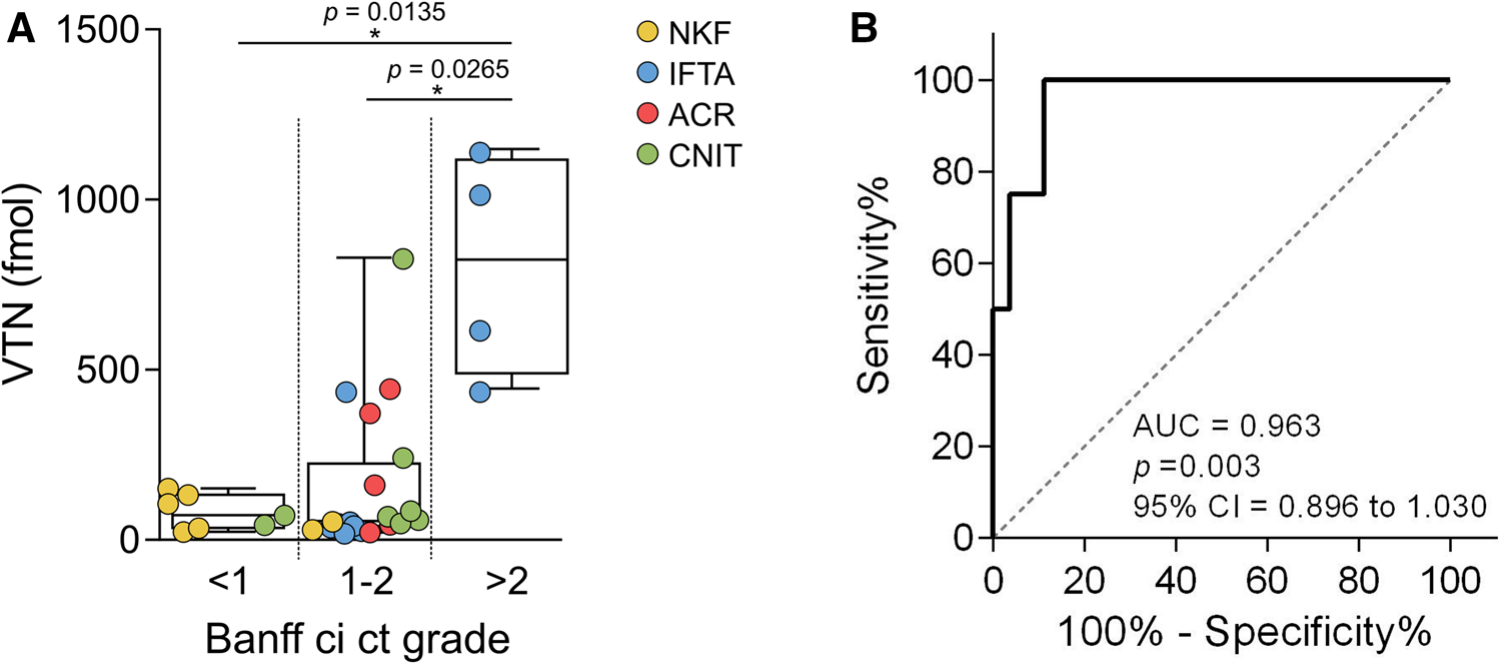
Vitronectin expression differences in uEVs measured by targeted MS regarding kidney fibrosis grade. **a** Vitronectin expression in uEVs by targeted mass spectrometry regarding the Banff criteria of chronic interstitial and tubular lesions (ci ct) grade. The colour code indicates sample group. Boxplots show the mean of each group and whiskers show minimum to maximum. **b** ROC curve based on targeted proteomics levels of vitronectin as a stand-alone biomarker to differentiate Banff ci ct grades ≤ 2 (n = 27) from > 2 (n = 4). AUC, area under the curve; CI, confidence interval

Up to five proteins that showed a higher expression in pathological samples compared to NKF samples in the discovery phase were confirmed by targeted proteomics: CTSD, RBP4, VTN, CST3 and SERPINC1. When analysing the results in each particular pathological condition, ACR samples showed statistically significant differences with the NKF group for the expression of CTSD, RBP4 and SERPINC1 (Fig. 4). The IFTA and CNIT groups did not show significant differences either with the NKF group or the other pathological groups. However, we observed that RBP4 expression in the ACR group and vitronectin expression in the IFTA group showed a bimodal distribution of the samples (Hartigan’s dip test for unimodality *p*-value = 0.009 and 0.016, respectively). To dive more deeply into these observations, we further analysed 15 additional parameters of these samples, such as the origin of the organ (living or deceased donor), time from transplantation to collection of the sample, serum creatinine at sample collection, graft failure or induction drugs, among others. While no significant differences could be found between the two subgroups of ACR samples regarding the expression of RBP4, we observed that samples with the highest expression of vitronectin in the IFTA group were those that presented the highest degrees of chronic interstitial lesions and chronic tubular lesions in the histopathological results. Interestingly, the expression of vitronectin was significantly higher in patients showing a ci ct score > 2 or above, regardless of the pathological group of the sample (Fig. 5a). A receiver operating characteristics (ROC) curve to discriminate patients with ci ct score of ≤ 2 from > 2 presented an area under the curve (AUC) of 0.96 (Fig. 5b).

### Validation with ELISA

**Fig. 6:**
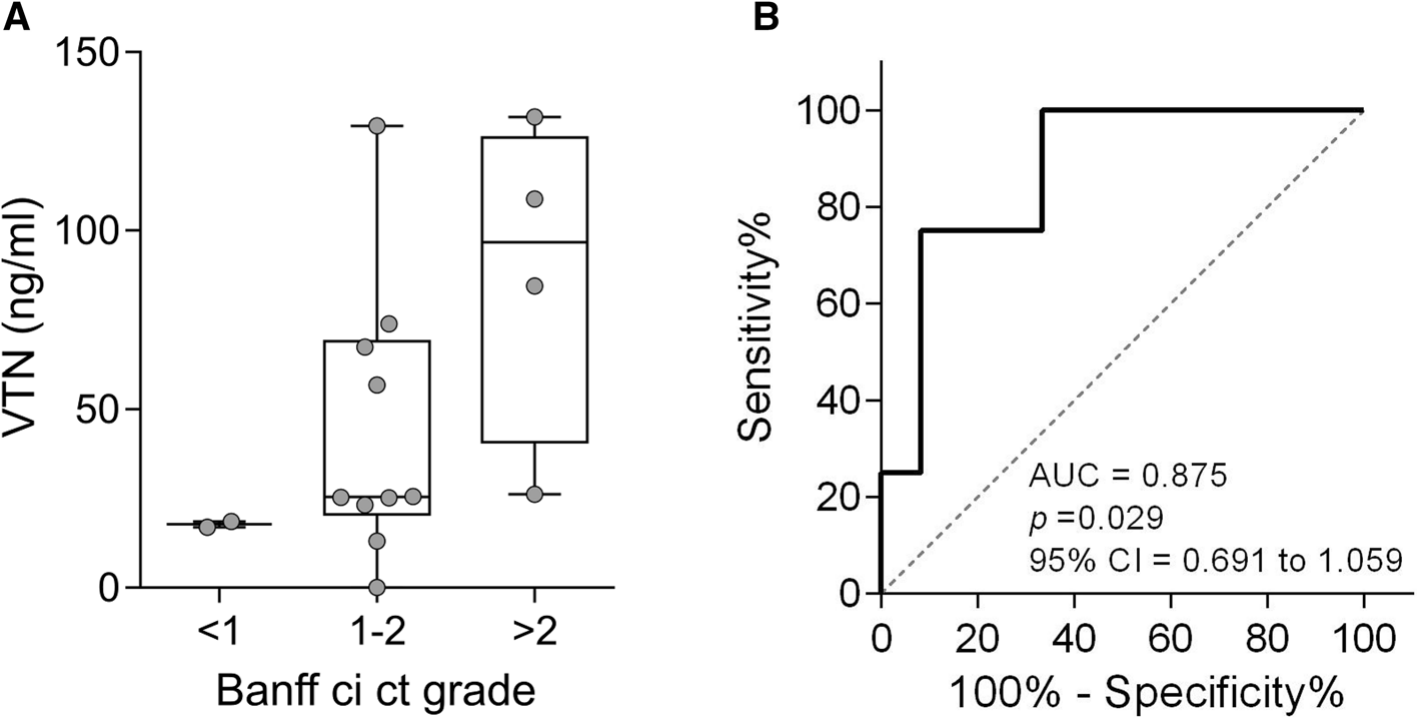
Vitronectin levels in concentrated urine measured by ELISA regarding kidney fibrosis grade. **a** Vitronectin concentration in urine was measured by ELISA and the values were stratified regarding the Banff criteria of chronic interstitial and tubular lesions (ci ct) grade. Boxplots show the mean of each group and whiskers show minimum to maximum. **b** ROC curve based on ELISA of vitronectin as a stand-alone biomarker to differentiate Banff ci ct grades ≤ 2 (n = 12) from > 2 (n = 4). AUC, area under the curve; CI, confidence interval

Since it is not feasible to apply mass spectrometry techniques to the clinical setting, the results related to vitronectin were further validated with a preliminary ELISA in urine samples from a limited number of kidney-transplanted patients with different grades of fibrosis. The results show that, similarly to the verification phase, samples from patients with a ci ct score of > 2 tended to have a higher concentration of vitronectin. Samples from patients with 1–2 ci ct score presented a high variation in their vitronectin concentration in urine. The two samples with a ci ct score of 0, presented a negligible vitronectin concentration (Fig. 6a). Similarly to the targeted proteomics results, a ROC curve of the ELISA results showed an AUC of 0.87 (Fig. 6b).

## Discussion

Accurate and early diagnosis of potential alterations in renal-transplanted patients is fundamental to improve both graft and patients’ survival. The current diagnostic method to determine the nature of the pathology is renal biopsy. This method is highly invasive, it cannot be performed frequently and it only analyses a small random portion of the kidney, what along with a rather subjective evaluation leads to around 20% of misdiagnosis [18, 21]. Knowing the potential of EV in the field of biomarker discovery and especially urinary EV for kidney-related pathologies, [68–70] we searched for protein biomarkers of kidney graft alterations in transplant recipients with four different diagnoses. Using a bottom-up proteomics approach in two independent cohorts, we found some EV-associated proteins differently expressed in patients with pathological kidneys compared to NKF patients. Moreover, we discovered vitronectin as a potential biomarker of kidney fibrosis.

Extracellular vesicles shuttle their bioactive cargo between cells and have a key role in many pathophysiological processes [71, 72]. Their paramount role in kidney transplantation rejection has been described in antibody-mediated rejection [73] and ischemia–reperfusion injury [74]. Furthermore, some studies envisage the use of EVs derived from mesenchymal stem/stromal cells (MSC) in the kidney transplant rejection setting, and report that they could inhibit tubular cell apoptosis and interstitial fibrosis, and promote proliferation of progenitor cells [75–78].

Focusing on the potential of EVs as biomarkers, in our study up to 777 proteins were confidently identified, which were significantly related to exosomes and extracellular-related terms. Interestingly, the NKF group had a lower number of identified proteins. In addition, we found several proteins differentially expressed in the uEVs from pathological samples compared to NKF. This suggests that pathological changes occurring in the kidney are reflected in the proteomic content of uEVs [69, 70].

At the beginning of the study, patients of the two cohorts were classified exclusively based on histopathological diagnosis and clinical criteria into NKF, IFTA, ACR and CNIT groups. Although distinctive diagnosis for CNIT or IFTA is controversial, the PCA analyses of proteomic data clearly segregated IFTA from CNIT samples, pointing to a different proteomic pattern between these two entities. One of the most interesting proteins identified in our study was vitronectin, which was significantly more expressed in patients with a high degree of kidney fibrosis–determined by ci ct Banff score–in the verification phase. Vitronectin is a protein with multiple roles found in the serum and in the extracellular matrix. In the lower urinary tract, vitronectin can originate from plasma extravasation and from synthesis by the urothelium [43], but it is difficult to determine the precedence of the uEV-related vitronectin we found in our samples. One of the functions of vitronectin is to inhibit the terminal complement pathway and the membrane attach complex (MAC). [46] Vitronectin is found in sclerotic glomeruli in immune deposits containing C5b-9, [79] and one study suggests that it plays a protective role in the glomeruli in membranous nephropathy [80]. Conversely, it binds with high affinity to PAI-1, a potent profibrotic glycoprotein, and their binding plays a key role in the development of fibrosis in several tissues, although the exact mechanisms are controversial. In the kidney, it has been described to increase PAI-1 activity and hamper fibrinolysis leading to an aggravation of renal failure [81]. Moreover, in the event of vascular injury, PAI-1 and vitronectin promote neointima formation after vascular injury [82]. Contrarily, in another study with a mouse cardiac fibrosis model, the results showed that binding of vitronectin with PAI-1 could be protective against fibrosis [46]. Other studies also support that vitronectin inhibits fibrogenesis by interacting with PAI-1 [83, 84], while others yet state that it has no effect [85, 86]. In either case, vitronectin promises to be not only an interesting diagnosis biomarker but also a therapeutic target for kidney fibrosis that warrants further investigation.

Vitronectin also participates in blood coagulation by interacting with SERPINC1 [44]. Both proteins were found more expressed in pathological samples and the GO term “Regulation of protein activation cascade”, was also enriched in the pathological groups. Conversely to what is observed with vitronectin, we did not find a direct association between SERPINC1 and high levels of fibrosis.

Changes in the levels of urinary vitronectin have been reported before. In a study by Takahashi et al. [87] vitronectin was investigated by ELISA in the urine of paediatric kidney patients. The protein was significantly increased when mesangial sclerosis changes were on-going. Furthermore, in a murine model, kidney vitronectin mRNA and protein expression was higher in mice with chronic kidney disease than in controls [85], yet the same authors also found that vitronectin did not have an implication in fibrogenesis in a Vtn knock-out mice model.

With regard to the fibrotic level of kidneys, Mohammed-Ali Zahraa et al. [88] recently reported six angiotensin II-related proteins that were increased in patients diagnosed with mild fibrosis compared with absence of fibrosis. However, they could not distinguish between moderate or severe fibrosis. Noticeably, moderate to severe fibrosis has been associated with poorer prognosis regarding renal function and graft survival [28–37], therefore stressing the importance of a biomarker for such stages. While our results showed that vitronectin values in targeted proteomics clearly discriminate between low (< 1) and severe (> 2) levels of fibrosis, we also detected some overlapping in the expression of vitronectin between patients with low-moderate (1–2) and severe (> 2) grade of fibrosis. This overlapping may be due to several reasons, including a possible misclassification of the samples due to the sampling and the evaluation of the renal biopsy – which is estimated to be around 20% [18, 21]. Of note, 20% (4 out of 20) of samples in the low-moderate fibrosis group (1–2) were those contributing to higher deviation. Although vitronectin was among the significant proteins found in the discovery phase, none of the patients in this phase presented grade3 fibrosis, and therefore a limitation of the study may be that the relationship of vitronectin with grade 3 fibrosis could only be observed in the second phase. Yet, as targeted mass spectrometry is still far from being applicable to the clinical setting, we also investigated whether our results were similar in a conventional ELISA, a cost-efficient, fast, simple and sensitive method for the detection of proteins. In a pilot study, with a still limited number of patients, ELISA revealed similar results to targeted proteomics data, thus validating the observation and confirming the translationality of the assay. Since the number of samples is still limited, further validation analyses on bigger cohorts of patients with different degrees of fibrosis will be performed to fully confirm these observations. Moreover, another limitation of our study was that most of the biopsies were performed before the newest Banff classification introduced the i-IFTA item [22]. Inflammation within areas of fibrosis is associated with cellular rejection, either acute or chronic, and worse outcomes (DeKAF cohort study) [89]. In the future, it would be interesting to analyse whether inflammation within areas of fibrosis has any effect on urinary proteomics.

For what we think is the first time, we report vitronectin as a potential non-invasive biomarker of severe kidney fibrosis in kidney-transplanted patients. When further validated in a larger cohort of patients, this observation may allow (1) more frequent monitoring of kidney transplanted patients, (2) sparing kidney biopsy and its associated complications, (3) an earlier diagnosis and (4) the possibility to apply the optimal treatment to regulate the progression of fibrosis. In this sense, severe fibrosis can only be slowed down and is still not reversible. However, anti-fibrotic therapies are under the spotlight of researchers, with currently more than 20 clinical studies on anti-fibrotic interventions in kidney (reviewed in [28]). All these would directly impact graft and patients’ survival, as well as improving their quality of life.

## Electronic supplementary material

Below is the link to the electronic supplementary material.

Supplementary file1 (DOCX 25 kb)

Supplementary file2 (DOCX 29 kb)

Supplementary file3 (DOCX 19 kb)

Supplementary file4 (DOCX 30 kb)

Supplementary file5 (DOCX 20 kb)

Supplementary file6 (PDF 254 kb)

Supplementary file7 (PDF 34 kb)

## Data Availability

Authors elected not to share data.
