# Supplementary material for: Urinary vitronectin identifies patients with high levels of fibrosis in kidney grafts
Source: J Nephrol. 2020 Dec 4;34(3):861–74. doi: 10.1007/s40620-020-00886-y (PMC8192319; doi:10.1007/s40620-020-00886-y)
Supplement: Supplementary file 1 — Supplementary file1 (DOCX 25 kb) [file 40620_2020_886_MOESM1_ESM.docx]

Supplementary table 1. Banff scoring results of the histopathological analysis of kidney biopsies from patients of the discovery cohort.

| **Group** | **Sample** | **i** | **t** | **v** | **g** | **ah** | **ci** | **ct** | **cv** | **cg** | **mm** | **ptc** | **ti** |
| --- | --- | --- | --- | --- | --- | --- | --- | --- | --- | --- | --- | --- | --- |
| IFTA | D_8 | 0 | 0 | 0 | 0 | 0 | 1 | 1 | 1 | 0 | 0 | 0 | 2 |
|  | D_9 | 0 | 0 | 0 | 0 | 3 | 1 | 1 | 1 | 0 | 0 | 0 | 1 |
|  | D_10 | 0 | 0 | 0 | 1 | 1 | 1 | 1 | 0 | 0 | 1 | 0 | 1 |
|  | D_11 | 0 | 0 | 0 | 0 | 3 | 2 | 2 | 3 | 0 | 1 | 0 | 1 |
|  | D_12 | 0 | 0 | 0 | 0 | 3 | 2 | 2 | 1 | 0 | 1 | 0 | 1 |
| ACR | D_13 | 0 | 0 | 0 | 0 | 3 | 2 | 2 | 3 | 0 | 0 | 0 | 2 |
|  | D_14 | 1 | 1 | 0 | 0 | 2 | 1 | 1 | 2 | 0 | 0 | 0 | 1 |
|  | D_15 | 3 | 2 | 1 | 1 | 0 | 0 | 0 | 0 | 0 | 0 | 3 | 3 |
|  | D_16 | 3 | 3 | 2 | 0 | 1 | 0 | 0 | 2 | 0 | 0 | 0 | 3 |
|  | D_17 | 2 | 2 | 2 | 0 | 3 | 1 | 1 | 2 | 0 | 1 | 0 | 2 |
|  | D_18 | 2 | 2 | 0 | 0 | 0 | 0 | 0 | 1 | 0 | 0 | 0 | 2 |
| CNIT | D_19 | 0 | 0 | 0 | 0 | 0 | 0 | 0 | 2 | 0 | 0 | 0 | 1 |
|  | D_20 | 0 | 0 | 0 | 0 | 0 | 1 | 1 | 0 | 0 | 0 | 0 | 1 |
|  | D_21 | 0 | 0 | 0 | 0 | 3 | 2 | 2 | 1 | 0 | 0 | 0 | 3 |
|  | D_22 | 0 | 0 | 0 | 0 | 0 | 1 | 1 | 1 | 0 | 1 | 0 | 0 |
|  | D_23 | 0 | 0 | 0 | 0 | 0 | 0 | 0 | 1 | 0 | 0 | 0 | 0 |

i, interstitial infiltrate; t, tubulitis; v, vasculitis; g, glomerulitis; ah, arteriolar hyalinosis; ci, chronic interstitial lesions; ct, chronic tubular lesions; cv, chronic vascular lesions; cg, chronic glomerular lesions; mm, mesangial matrix; ptc, peritubular capillaritis; ti, total interstitial inflammation.
