# Supplementary material for: Urinary vitronectin identifies patients with high levels of fibrosis in kidney grafts
Source: J Nephrol. 2020 Dec 4;34(3):861–74. doi: 10.1007/s40620-020-00886-y (PMC8192319; doi:10.1007/s40620-020-00886-y)
Supplement: Supplementary file 2 — Supplementary file2 (DOCX 29 kb) [file 40620_2020_886_MOESM2_ESM.docx]

Supplementary table 2. Banff scoring results of the histopathological analysis of kidney biopsies from patients of the verification cohort.

| **Group** | **Sample** | **i** | **t** | **v** | **g** | **ah** | **ci** | **ct** | **cv** | **cg** | **mm** | **ptc** | **ti** |
| --- | --- | --- | --- | --- | --- | --- | --- | --- | --- | --- | --- | --- | --- |
| NKF | V_1 | 0 | 0 | 0 | 0 | 0 | 0 | 1 | 0 | 0 | 0 | 0 | 0 |
|  | V_2 | 0 | 0 | 0 | 0 | 0 | 1 | 1 | 0 | 0 | 0 | 0 | 0 |
|  | V_3 | 0 | 0 | 0 | 0 | 0 | 0 | 0 | 0 | 0 | 0 | 0 | 0 |
|  | V_4 | 0 | 0 | 0 | 0 | 0 | 0 | 0 | 0 | 0 | 0 | 0 | 0 |
|  | V_5 | / | / | / | / | / | / | / | / | / | / | / | / |
|  | V_6 | 0 | 0 | 0 | 0 | 0 | 0 | 0 | 1 | 0 | 0 | 0 | 0 |
|  | V_7 | 0 | 0 | 0 | 0 | 1 | 1 | 1 | 0 | 0 | 0 | 0 | 1 |
|  | V_8 | 0 | 0 | 0 | 0 | 0 | 0 | 1 | 0 | 0 | 0 | 0 | 0 |
|  | V_9 | 0 | 0 | 0 | 0 | 0 | 0 | 0 | 0 | 0 | 0 | 0 | 0 |
|  | V_10 | / | / | / | / | / | / | / | / | / | / | / | / |
| IFTA | V_11 | 0 | 0 | 0 | 0 | 0 | 3 | 3 | 1 | 0 | 0 | 0 | 1 |
|  | V_12 | 0 | 0 | 0 | 0 | 2 | 3 | 3 | 1 | 0 | 2 | 0 | 1 |
|  | V_13 | 1 | 0 | 0 | 0 | 0 | 3 | 3 | 0 | 0 | 0 | 0 | 1 |
|  | V_14 | 0 | 0 | 0 | 0 | 0 | 3 | 2 | 2 | 0 | 0 | 0 | 1 |
|  | V_15 | 0 | 0 | 0 | 0 | 0 | 2 | 2 | 1 | 0 | 0 | 1 | 0 |
|  | V_16 | 0 | 0 | 0 | 0 | 0 | 2 | 2 | 1 | 0 | 0 | 2 | 1 |
|  | V_17 | 0 | 0 | 0 | 0 | 0 | 1 | 1 | 1 | 0 | 0 | 0 | 1 |
|  | V_18 | 0 | 0 | 0 | 0 | 1 | 2 | 2 | 1 | 0 | 0 | 0 | 0 |
|  | V_19 | 1 | 0 | 0 | 1 | 1 | 2 | 2 | 2 | 0 | 0 | 1 | 2 |
|  | V_20 | 0 | 0 | 0 | 0 | 1 | 2 | 2 | 2 | 0 | 0 | 0 | 1 |
|  | V_21 | 0 | 0 | 0 | 0 | 0 | 2 | 2 | 1 | 0 | 0 | 0 | 1 |
| ACR | V_22 | / | / | / | / | / | / | / | / | / | / | / | / |
|  | V_23 | 2 | 2 | 0 | 0 | 0 | 1 | 1 | 1 | 0 | 0 | 0 | 2 |
|  | V_24 | 3 | 3 | 0 | 0 | 1 | 1 | 1 | 2 | 0 | 0 | 0 | 0 |
|  | V_25 | 2 | 2 | 0 | 0 | 0 | 1 | 1 | 0 | 0 | 0 | 0 | 0 |
|  | V_26 | 2 | 3 | 0 | 0 | 0 | 2 | 1 | 1 | 0 | 0 | 1 | 2 |
|  | V_27 | 1 | 3 | 0 | 0 | 0 | 1 | 1 | 1 | 0 | 0 | 1 | 1 |
|  | V_28 | 1 | 1 | 0 | 0 | 0 | 0 | 0 | 0 | 0 | 0 | 0 | 1 |
|  | V_29 | / | / | / | / | / | / | / | / | / | / | / | / |
|  | V_30 | 3 | 3 | 0 | 0 | 1 | 1 | 1 | 1 | 0 | 0 | 1 | 3 |
|  | V_31 | 2 | 2 | 0 | 0 | 0 | 1 | 1 | 0 | 0 | 0 | 0 | 2 |
| CNIT | V_32 | 0 | 0 | 0 | 0 | 3 | 2 | 2 | 2 | 0 | 1 | 0 | 1 |
|  | V_33 | 0 | 0 | 0 | 1 | 3 | 2 | 2 | 2 | 0 | 0 | 0 | 1 |
|  | V_34 | 0 | 0 | 0 | 0 | 0 | 1 | 0 | 1 | 0 | 0 | 0 | 0 |
|  | V_35 | 0 | 0 | 0 | 0 | 2 | 2 | 2 | 1 | 0 | 0 | 0 | 0 |
|  | V_36 | / | / | / | / | / | / | / | / | / | / | / | / |
|  | V_37 | 0 | 0 | 0 | 0 | 0 | 0 | 1 | 1 | 0 | 0 | 0 | 0 |
|  | V_38 | 0 | 0 | 0 | 0 | 1 | 1 | 1 | 0 | 0 | 0 | 0 | 1 |
|  | V_39 | / | / | / | / | / | / | / | / | / | / | / | / |
|  | V_40 | 0 | 0 | 0 | 0 | 0 | 2 | 2 | 2 | 0 | 0 | 0 | 1 |
|  | V_41 | 0 | 0 | 0 | 0 | 1 | 1 | 1 | 0 | 0 | 0 | 0 | 1 |

i, interstitial infiltrate; t, tubulitis; v, vasculitis; g, glomerulitis; ah, arteriolar hyalinosis; ci, chronic interstitial lesions; ct, chronic tubular lesions; cv, chronic vascular lesions; cg, chronic glomerular lesions; mm, mesangial matrix; ptc, peritubular capillaritis; ti, total interstitial inflammation. In six cases, a complete Banff score could not be carried out because of insufficient material.
