# Supplementary material for: Urinary vitronectin identifies patients with high levels of fibrosis in kidney grafts
Source: J Nephrol. 2020 Dec 4;34(3):861–74. doi: 10.1007/s40620-020-00886-y (PMC8192319; doi:10.1007/s40620-020-00886-y)
Supplement: Supplementary file 3 — Supplementary file3 (DOCX 19 kb) [file 40620_2020_886_MOESM3_ESM.docx]

Supplementary table 3. List of the 15 proteins shared by all samples

| **Gene names** | **Protein names** |
| --- | --- |
| EZR | Ezrin |
| IGHG1 | Ig gamma-1 chain C region |
| IGHG3 | Ig gamma-3 chain C region |
| IGLL5;IGLC1 | Immunoglobulin lambda-like polypeptide 5;Ig lambda-1 chain C regions |
| IGKV3-11;IGKC;IGKV1-8 | Ig kappa chain C region |
| UBB;RPS27A;UBC;UBA52;UBBP4 | Ubiquitin-60S ribosomal protein L40;Ubiquitin;60S ribosomal protein L40;Ubiquitin-40S ribosomal protein S27a;Ubiquitin;40S ribosomal protein S27a;Polyubiquitin-B;Ubiquitin;Polyubiquitin-C;Ubiquitin |
| CD59 | CD59 glycoprotein |
| IGHA1 | Ig alpha-1 chain C region |
| AMBP | Protein AMBP;Alpha-1-microglobulin;Inter-alpha-trypsin inhibitor light chain;Trypstatin |
| GAPDH | Glyceraldehyde-3-phosphate dehydrogenase |
| UMOD | Uromodulin;Uromodulin, secreted form |
| HSPA8 | Heat shock cognate 71 kDa protein |
| ANPEP | Aminopeptidase N |
| DEFA3;DEFA1 | Neutrophil defensin 3;HP 3-56;Neutrophil defensin 2;Neutrophil defensin 1;HP 1-56;Neutrophil defensin 2 |
| PPIA | Peptidyl-prolyl cis-trans isomerase A;Peptidyl-prolyl cis-trans isomerase A, N-terminally processed;Peptidyl-prolyl cis-trans isomerase |
