# Supplementary material for: Urinary vitronectin identifies patients with high levels of fibrosis in kidney grafts
Source: J Nephrol. 2020 Dec 4;34(3):861–74. doi: 10.1007/s40620-020-00886-y (PMC8192319; doi:10.1007/s40620-020-00886-y)
Supplement: Supplementary file 4 — Supplementary file4 (DOCX 30 kb) [file 40620_2020_886_MOESM4_ESM.docx]

Supplementary table 4. Volcano plot full list of significant proteins in the discovery phase. Proteins more expressed in the Pathological groups have a positive fold change, whereas proteins more expressed in the NKF group have a negative fold change (last seven proteins).

| p-value | Fold Change | Majority protein IDs | Protein names | Gene names |  |
| --- | --- | --- | --- | --- | --- |
| 4.80E-08 | 17.157 | Q5VY30;A0A0C4DGV7;P02753 | Retinol-binding protein 4;Plasma retinol-binding protein(1-182);Plasma retinol-binding protein(1-181);Plasma retinol-binding protein(1-179);Plasma retinol-binding protein(1-176) | RBP4 |  |
| 4.83E-08 | 17.861 | P07339;C9JH19;H7C469;F8WD96 | Cathepsin D;Cathepsin D light chain;Cathepsin D heavy chain | CTSD |  |
| 9.75E-06 | 16.235 | P01008;Q8TCE1 | Antithrombin-III | SERPINC1 |  |
| 1.07E-05 | 17.709 | P02766;A0A087WT59;A0A087WV45 | Transthyretin | TTR |  |
| 1.38E-05 | 15.238 | P02671 | Fibrinogen alpha chain;Fibrinopeptide A;Fibrinogen alpha chain | FGA |  |
| 1.01E-04 | 14.252 | P04004 | Vitronectin;Vitronectin V65 subunit;Vitronectin V10 subunit;Somatomedin-B | VTN |  |
| 1.29E-04 | 13.613 | P18669;P15259 | Phosphoglycerate mutase 1;Phosphoglycerate mutase 2 | PGAM1;PGAM2 |  |
| 1.37E-04 | 13.024 | P06744;K7EQ48;A0A0A0MTS2;A0A0J9YXP8;A0A0J9YYH3;A0A0J9YX90 | Glucose-6-phosphate isomerase | GPI |  |
| 1.70E-04 | 10.905 | P26038 | Moesin | MSN |  |
| 2.10E-04 | 14.795 | P14174 | Macrophage migration inhibitory factor | MIF |  |
| 2.35E-04 | 15.513 | P00915;E5RHP7;E5RH81;E5RFE7;E5RIF9;E5RG43;H0YBE2 | Carbonic anhydrase 1 | CA1 |  |
| 2.87E-04 | 12.983 | P16152;E9PQ63;A8MTM1 | Carbonyl reductase [NADPH] 1 | CBR1 |  |
| 3.39E-04 | 11.930 | P14618;B4DNK4;H3BTN5;H3BR70;H3BQ34 | Pyruvate kinase PKM;Pyruvate kinase | PKM |  |
| 4.00E-04 | 15.883 | P01034 | Cystatin-C | CST3 |  |
| 8.13E-04 | 10.923 | Q96IU4;F8W9U3;B4DQI4 | Alpha/beta hydrolase domain-containing protein 14B | ABHD14B |  |
| 1.09E-03 | 11.564 | B7ZKJ8;Q14624;H7C0L5 | Inter-alpha-trypsin inhibitor heavy chain H4;70 kDa inter-alpha-trypsin inhibitor heavy chain H4;35 kDa inter-alpha-trypsin inhibitor heavy chain H4 | ITIH4 |  |
| 1.17E-03 | 11.906 | B4E1Z4;E7ETN3;P00751 | Complement factor B;Complement factor B Ba fragment;Complement factor B Bb fragment | CFB |  |
| 1.26E-03 | 10.247 | P61006 | Ras-related protein Rab-8A | RAB8A |  |
| 1.27E-03 | 12.670 | P09211;A8MX94 | Glutathione S-transferase P | GSTP1 |  |
| 1.39E-03 | 12.335 | P62834;A0A075B6Q0 | Ras-related protein Rap-1A | RAP1A |  |
| 1.58E-03 | 11.140 | P27348;E9PG15 | 14-3-3 protein theta | YWHAQ |  |
| 1.75E-03 | 12.589 | O14773;A0A0C4DGZ9 | Tripeptidyl-peptidase 1 | TPP1 |  |
| 1.77E-03 | 12.035 | P60174 | Triosephosphate isomerase | TPI1 |  |
| 1.87E-03 | 10.708 | O43707 | Alpha-actinin-4 | ACTN4 |  |
| 1.94E-03 | 12.870 | O96009;M0QXC5 | Napsin-A | NAPSA |  |
| 2.00E-03 | 11.651 | Q9Y696 | Chloride intracellular channel protein 4 | CLIC4 |  |
| 2.10E-03 | 13.422 | P01019 | Angiotensinogen;Angiotensin-1;Angiotensin-2;Angiotensin-3;Angiotensin-4;Angiotensin 1-9;Angiotensin 1-7;Angiotensin 1-5;Angiotensin 1-4 | AGT |  |
| 3.45E-03 | 11.347 | P40925;B9A041;B8ZZ51;C9JF79 | Malate dehydrogenase, cytoplasmic;Malate dehydrogenase | MDH1 |  |
| 3.79E-03 | 9.934 | Q14344 | Guanine nucleotide-binding protein subunit alpha-13 | GNA13 |  |
| 3.90E-03 | 11.205 | Q5JP53;P07437;Q5ST81;Q9BVA1;Q13885 | Tubulin beta chain;Tubulin beta-2B chain;Tubulin beta-2A chain | TUBB;TUBB2B;TUBB2A |  |
| 4.00E-03 | 8.969 | Q9UHR4 | Brain-specific angiogenesis inhibitor 1-associated protein 2-like protein 1 | BAIAP2L1 |  |
| 4.06E-03 | 10.758 | Q9UBR2 | Cathepsin Z | CTSZ |  |
| 4.16E-03 | 10.090 | Q12929 | Epidermal growth factor receptor kinase substrate 8 | EPS8 |  |
| 4.26E-03 | 10.721 | Q9UHL4 | Dipeptidyl peptidase 2 | DPP7 |  |
| 4.33E-03 | 9.331 | A0A087WUL0;Q3LXA3;H0YCY6 | Bifunctional ATP-dependent dihydroxyacetone kinase/FAD-AMP lyase (cyclizing);ATP-dependent dihydroxyacetone kinase;FAD-AMP lyase (cyclizing) | TKFC;DAK |  |
| 4.35E-03 | 10.073 | Q14914;Q5JVP2 | Prostaglandin reductase 1 | PTGR1 |  |
| 4.85E-03 | 11.540 | P02748 | Complement component C9;Complement component C9a;Complement component C9b | C9 |  |
| 4.99E-03 | 9.234 | A0A0G2JIW1;P0DMV9;P0DMV8;V9GZ37 | Heat shock 70 kDa protein 1B;Heat shock 70 kDa protein 1A | HSPA1B;HSPA1A |  |
| 6.27E-03 | 10.736 | Q5T2W1;A8MUH7 | Na(+)/H(+) exchange regulatory cofactor NHE-RF3;Putative PDZ domain-containing protein 1P | PDZK1;PDZK1P1 |  |
| 7.32E-03 | 8.916 | P01024 | Complement C3;Complement C3 beta chain;C3-beta-c;Complement C3 alpha chain;C3a anaphylatoxin;Acylation stimulating protein;Complement C3b alpha chain;Complement C3c alpha chain fragment 1;Complement C3dg fragment;Complement C3g fragment;Complement C3d fragment;Complement C3f fragment;Complement C3c alpha chain fragment 2 | C3 |  |
| 9.16E-03 | 8.514 | P10909;H0YC35;H0YLK8 | Clusterin;Clusterin beta chain;Clusterin alpha chain;Clusterin | CLU |  |
| 9.35E-03 | 10.289 | J3KRE2;J3KTF8;J3QQX2;P52565;J3KS60;J3KRY1 | Rho GDP-dissociation inhibitor 1 | ARHGDIA |  |
| 9.36E-03 | 8.810 | A0A087X0K1;Q9Y376 | Calcium-binding protein 39 | CAB39 |  |
| 9.39E-03 | 9.579 | P00558 | Phosphoglycerate kinase 1 | PGK1 |  |
| 9.52E-03 | 9.659 | C9J7K9;O15162;H7C5I5;C9J9P4 | Phospholipid scramblase 1 | PLSCR1 |  |
| 9.53E-03 | 8.721 | O75955;A2AB09 | Flotillin-1 | FLOT1 |  |
| 9.67E-03 | 9.900 | B5MC82;P30046;A6NHG4;J3KQ18 | D-dopachrome decarboxylase;D-dopachrome decarboxylase-like protein | DDT;DDTL |  |
| 9.73E-03 | 7.884 | Q08257;A6NP24 | Quinone oxidoreductase | CRYZ |  |
| 2.47E-03 | -10.947 | Q02413 | Desmoglein-1 | DSG1 | |
| 3.36E-03 | -11.812 | P31431 | Syndecan-4 | SDC4 | |
| 3.38E-03 | -9.594 | Q96FE7;C9JMK5 | Phosphoinositide-3-kinase-interacting protein 1 | PIK3IP1 | |
| 3.39E-03 | -8.301 | Q5T749 | Keratinocyte proline-rich protein | KPRP | |
| 3.48E-03 | -8.234 | P09603;E9PJA2 | Macrophage colony-stimulating factor 1;Processed macrophage colony-stimulating factor 1 | CSF1 | |
| 6.06E-03 | -9.222 | Q08554 | Desmocollin-1 | DSC1 | |
| 7.76E-03 | -12.723 | P81605 | Dermcidin;Survival-promoting peptide;DCD-1 | DCD | |
