# Supplementary material for: Urinary vitronectin identifies patients with high levels of fibrosis in kidney grafts
Source: J Nephrol. 2020 Dec 4;34(3):861–74. doi: 10.1007/s40620-020-00886-y (PMC8192319; doi:10.1007/s40620-020-00886-y)
Supplement: Supplementary file 5 — Supplementary file5 (DOCX 20 kb) [file 40620_2020_886_MOESM5_ESM.docx]

Supplementary table 5. List of peptides used in targeted proteomics.

| **Gene name** | **NCBI Entry** | **Peptide sequence** |
| --- | --- | --- |
| ACE2 | Q9BYF1 | ISFNFFVTAP(K) |
|  |  | SGENPYASIDIS(K) |
| ACTR3 | P61158 | DYEEIGPSI[C](R) |
|  |  | LGYAGNTEPQFIIPS[C]IAI(K) |
| AFM | P43652 | ESLLNHFLYEVA(R) |
|  |  | IAPQLSTEELVSLGE(K) |
| BASP1 | P80723 | ESEPQAAAEPAEA(K) |
|  |  | ETPAATEAPSSTP(K) |
| C5 | P01031 | IDTQDIEASHY(R) |
|  |  | TDAPDLPEENQA(R) |
| C7 | P10643 | LIDQYGTHYLQSGSLGGEY(R) |
|  |  | NVVYT[C]NEGYSLIGNPVA(R) |
| CDC42 | P60953 | NVFDEAILAALEPPEP(K) |
|  |  | T[C]LLISYTTN(K) |
| CST3 | P01034 | ALDFAVGEYN(K) |
|  |  | TQPNLDN[C]PFHDQPHL(K) |
| CSTD | P07339 | LVDQNIFSFYLS(R) |
|  |  | VGFAEAA(R) |
| EZR | P15311 | FYPEDVAEELIQDITQ(K) |
|  |  | SQEQLAAELAEYTA(K) |
| IGFALS | P35858 | DLHFLEELQLGHN(R) |
|  |  | LHSLHLEGS[C]LG(R) |
| ISLR | O14498 | ALPGTPVASSQP(R) |
|  |  | TVAAGALASLSHL(K) |
| LUM | P51884 | NIPTVNENLENYYLEVNQLE(K) |
|  |  | SLEYLDLSFNQIA(R) |
| MB | P02144 | VEADIPGHGQEVLI(R) |
|  |  | YLEFISE[C]IIQVLQS(K) |
| OGN | P20774 | DFADIPNL(R) |
|  |  | LSLLEELSLAENQLL(K) |
| PPL | O60437 | EVVQEILQFQEDPQT(K) |
|  |  | SLLGEVEQNLQAA(K) |
| PROM1 | O43490 | AFTDLNSINSVLGGGILD(R) |
|  |  | VLPIEQSLSTLYQSV(K) |
| RBP4 | P02753 | DPNGLPPEAQ(K) |
|  |  | LIVHNGY[C]DG(R) |
| SERPINA4 | P29622 | LGFTDLFS(K) |
|  |  | VGSALFLSHNL(K) |
| SERPINC1 | P01008 | AFLEVNEEGSEAAASTAVVIAG(R) |
|  |  | TSDQIHFFFA(K) |
| TF | P02787 | D[C]HLAQVPSHTVVA(R) |
|  |  | DGAGDVAFV(K) |
| UPK1B | O75841 | EPLNLEA[C](K) |
|  |  | TENNDADYPWP(R) |
| VTN | P04004 | FEDGVLDPDYP(R) |
|  |  | GQY[C]YELDE(K) |
