# Supplementary material for: Urinary vitronectin identifies patients with high levels of fibrosis in kidney grafts
Source: J Nephrol. 2020 Dec 4;34(3):861–74. doi: 10.1007/s40620-020-00886-y (PMC8192319; doi:10.1007/s40620-020-00886-y)
Supplement: Supplementary file 6 — Supplementary file6 (PDF 254 kb) [file 40620_2020_886_MOESM6_ESM.pdf]

Fig S1

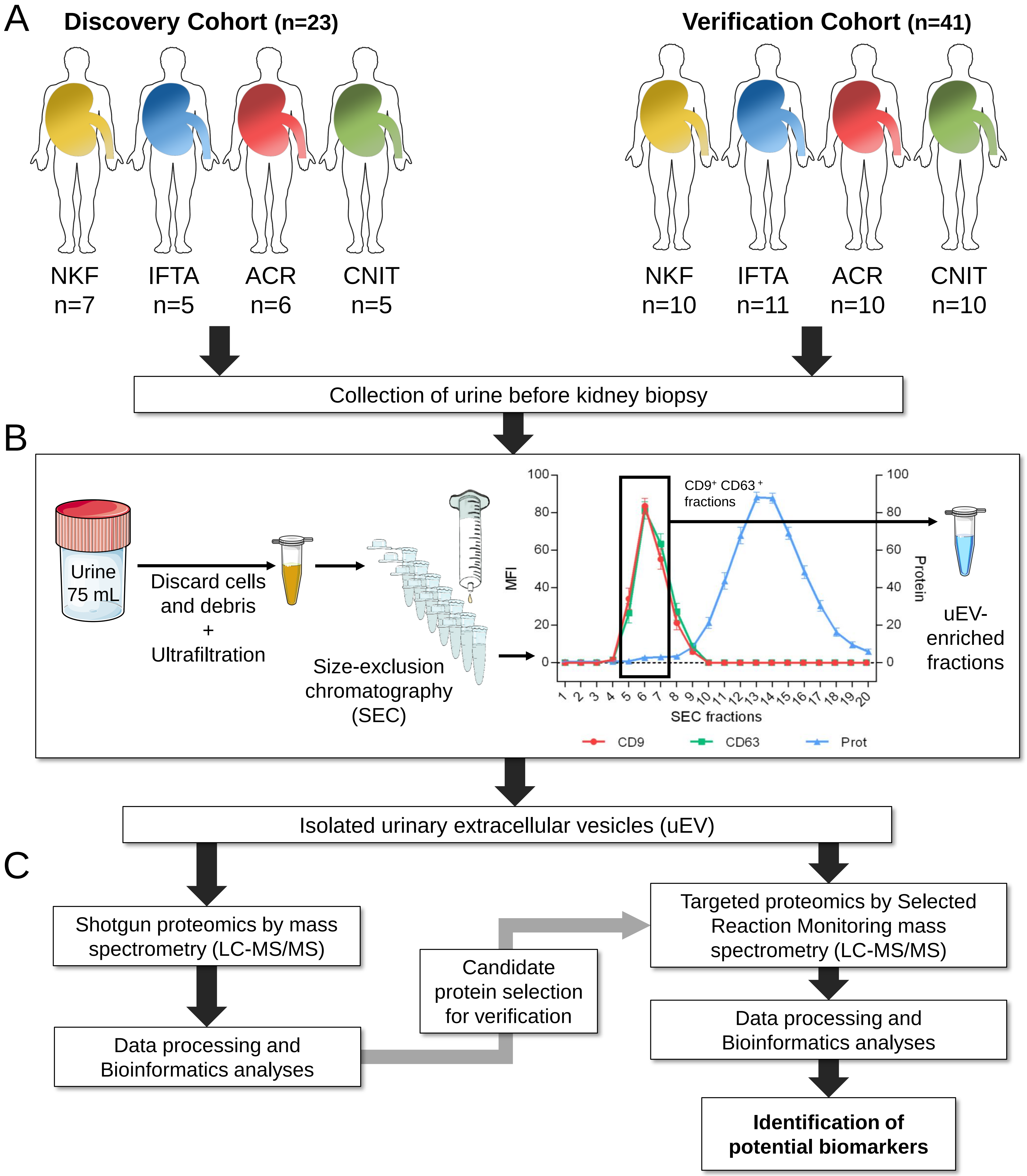
A
Discovery Cohort (n=23)
NKF n=7
IFTA n=5
ACR n=6
CNIT n=5
Verification Cohort (n=41)
NKF n=10
IFTA n=11
ACR n=10
CNIT n=10
Collection of urine before kidney biopsy
B
Urine 75 mL
Discard cells and debris + Ultrafiltration
Size-exclusion chromatography (SEC)
CD9+ CD63+ fractions
MFI
Protein
SEC fractions
CD9
CD63
Prot
uEV-enriched fractions
Isolated urinary extracellular vesicles (uEV)
C
Shotgun proteomics by mass spectrometry (LC-MS/MS)
Data processing and Bioinformatics analyses
Candidate protein selection for verification
Targeted proteomics by Selected Reaction Monitoring mass spectrometry (LC-MS/MS)
Data processing and Bioinformatics analyses
Identification of potential biomarkers
