# Supplementary figures and images for: Urinary vitronectin identifies patients with high levels of fibrosis in kidney grafts
Source: J Nephrol. 2020 Dec 4;34(3):861–74. doi: 10.1007/s40620-020-00886-y (PMC8192319; doi:10.1007/s40620-020-00886-y)

# Fig S2

—●—  $-\log(p\text{-value})$       —■—  $p\text{-value} = 0.05$

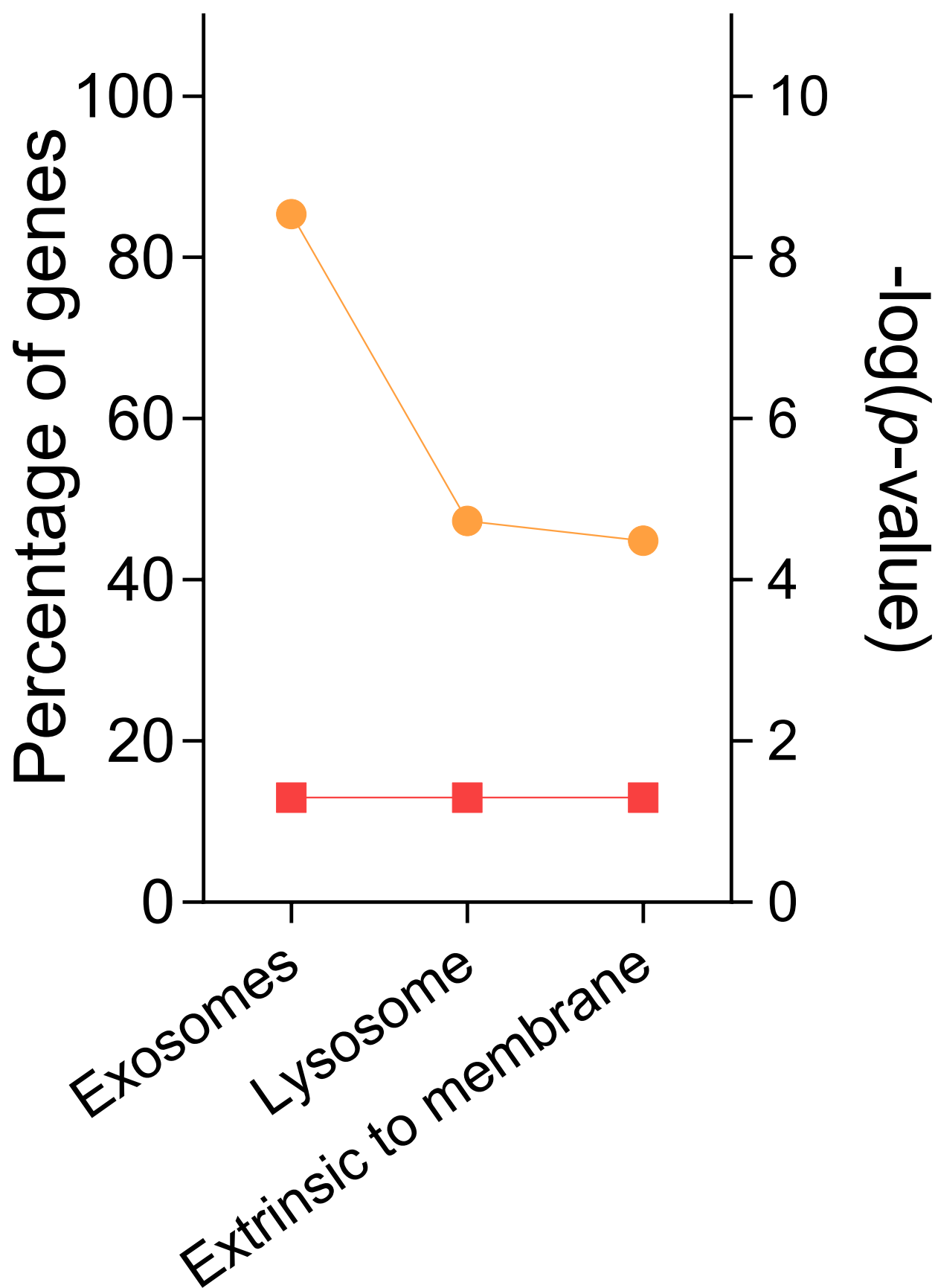

Supplement: Supplementary file 7 — Supplementary file7 (PDF 34 kb) [file 40620_2020_886_MOESM7_ESM.pdf]
